# Unveiling hidden molluscan diversity: New species records in Bougainville Bay, Strait of Magellan

**DOI:** 10.3897/BDJ.13.e157304

**Published:** 2025-09-05

**Authors:** Leslie Novoa, Cristian Aldea, Claudia Andrade, Belén Guarda, Jorge Holtheuer, Dirk Schories

**Affiliations:** 1 Departamento de Ciencias y Recursos Naturales, Facultad de Ciencias, Universidad de Magallanes, Punta Arenas, Chile Departamento de Ciencias y Recursos Naturales, Facultad de Ciencias, Universidad de Magallanes Punta Arenas Chile; 2 Programa de Magíster en Ciencias, Mención Manejo y Conservación de Recursos Naturales en Ambientes Subantárticos, Universidad de Magallanes, Punta Arenas, Chile Programa de Magíster en Ciencias, Mención Manejo y Conservación de Recursos Naturales en Ambientes Subantárticos, Universidad de Magallanes Punta Arenas Chile; 3 Centro de Investigación GAIA Antártica, Punta Arenas, Chile Centro de Investigación GAIA Antártica Punta Arenas Chile; 4 Laboratorio de Ecología Funcional, Instituto de la Patagonia, Universidad de Magallanes, Punta Arenas, Chile Laboratorio de Ecología Funcional, Instituto de la Patagonia, Universidad de Magallanes Punta Arenas Chile; 5 Wildlife Conservation Society Chile (WCS-Chile), Punta Arenas, Chile Wildlife Conservation Society Chile (WCS-Chile) Punta Arenas Chile; 6 KOJH Asesorías Ambientales, Punta Arenas, Chile KOJH Asesorías Ambientales Punta Arenas Chile; 7 German Aerospace Center (DLR), Köln, Germany German Aerospace Center (DLR) Köln Germany

**Keywords:** Mollusca, Magallanes, Brunswick Peninsula, Cape Froward, Global Biodiversity Information Facility, benthos

## Abstract

The Chilean Fjords and Channels ecoregion presents unique oceanographic conditions that are sensitive to environmental impacts and socio-ecological systems. To assess how these impacts affect these ecosystems, it is essential to examine the current status of highly diverse taxa, such as molluscs, in terms of biodiversity and abundance, as they serve as valuable macroecological indicators of marine ecosystem health. On the south-eastern margin of the Brunswick Peninsula, south of Punta Santa Ana, lies Cape Froward, where a system of eight small bays (< ca. 4 km²) is located, some of which are used as temporary harbours during navigation. Despite previous sampling efforts and existing records, these areas remain poorly described, with the region around Punta Santa Ana Bay being the most extensively documented. As part of monitoring programmes on coastal biodiversity in Magallanes and fishery resource assessments, sublittoral sampling was conducted in Bougainville Bay using autonomous diving (20 m depth) and a modified Agassiz trawl (44 m depth). This study recorded 49 operational taxonomic units, comprising 32 gastropods, nine bivalves and eight polyplacophorans, all of which represent previously unpublished diversity records for this Bay. Additionally, these species were categorised, based on their feeding strategies, providing insights into their ecological roles. These findings also extend to neighbouring bays along the southern Brunswick Peninsula (from El Águila Bay to Cape Froward), where only two sublittoral species — the bivalves *Zygochlamys patagonica* and *Philobrya sublaevis* — had been previously documented. In the central micro-basin of the Strait of Magellan (from Second Narrows to Carlos III Island), this study introduces six new records: one bivalve and five gastropods, including two nudibranchs. All findings were compared with data from the Global Biodiversity Information Facility. These newly-documented records are crucial for understanding the biodiversity of molluscs inhabiting frequently navigated maritime areas, serving as indicators of anthropogenic impacts. This is especially relevant given the recent proposal of the area as a National Park.

## Introduction

In the southeast Pacific, the Magallanes Fjords and Channels Biogeographic Ecoregion extends from the southern part of the Gulf of Penas to Cape Horn (48° to 56°S; Camus (2001), Spalding et al. (2007)). This unique ecosystem is where the Pacific and Atlantic Oceans converge, integrating different water masses and creating distinct features that shape marine life in this remote region (Fernández et al. 2000, Miloslavich et al. 2011). These ecological characteristics lead to significant variations in the composition, richness and structure of rocky coastal communities of benthic marine invertebrates compared to the rest of the temperate coasts of the Americas (Rosenfeld et al. 2013). Moreover, the Strait of Magellan acts as a natural passage between oceans and plays a crucial role in socio-ecological systems, evolving alongside human development. As an area of high maritime traffic, it also faces increasing pressures due to commercial interests in its marine resources.

These singularities, along with oceanographic characteristics, allow the Strait to be divided into three micro-basins: the eastern basin, located between the Atlantic Ocean entrance and Second Narrows; the central basin, extending towards Carlos III Island; and the western basin, leading to the Pacific Ocean entrance (Valdenegro and Silva 2003). These features have made it a subject of scientific study through naturalistic and oceanographic expeditions, beginning with the first expedition by King and Broderip (1832) and the last time with the *Cruceros de Investigación en Áreas Remotas* (CIMAR) (Comité Oceanográfico Nacional 2021).

This area did not always connect both oceans. Approximately 14,000 years BP, this flow was interrupted by large ice masses that later retreated. This glacial influence gave rise to climatic patterns typical of the southern tip of Patagonia, generating a gradient in the region’s glacial ecosystems (52°S; McCulloch et al. (2019)). Thus, glacier composition may be one of the most influential components of these ecosystems (Hodgson et al. 2014). The marine ecosystems of fjords and channels in these latitudes exhibit high seasonal primary productivity (Iriarte et al. 2001), largely due to stratification in the water column caused by freshwater inputs (Pizarro et al. 2006, Aracena et al. 2015).

The benthic communities of marine macroinvertebrates consist of organisms associated with the seafloor, either sessile or vagile. This characteristic makes them ideal biogeographical study models for observing ecosystem changes. Amongst the most abundant and diverse are molluscs, with around 85,000 described species (Chapman and Blockley 2009, Zhang 2011, Zhang 2013), most of which are found in marine environments (about 50,000 species). Along with crustaceans, molluscs form the most diverse group of marine invertebrates (Bouchet 2006, WoRMS Editorial Board 2025). They are particularly useful for faunistic biogeographic analyses, especially considering that they are one of the principal components of the benthic biota (Brandt et al. 1999, Linse et al. 2006).

Molluscs are found in various marine environments, exhibit diverse feeding habits and play an active role in ecosystems as both prey and predators (Haszprunar and Wanninger 2012). Additionally, they function as bioengineers in habitat formation, such as mussel beds (Kelaher et al. 2007). These characteristics make them valuable for environmental monitoring programmes, as they are taxonomically well-documented, particularly in the two most diverse classes, Bivalvia and Gastropoda, which serve as bioindicators of water quality and pollution (Oehlmann and Schulte-Oehlmann 2003).

Molluscs also display irregular distribution patterns associated with latitude. While biodiversity generally decreases towards higher latitudes, a considerable increase in mollusc diversity has been observed in southern Chile, beginning around 42°S (Valdovinos et al. 2003), near the northern boundary of the Magellanic Biogeographic Province (Spalding et al. 2007), where the Chilean fjords and channels begin. Habitat complexity promotes ecological interactions by increasing the number of niches, protection zones and trophic relationships (Kovalenko et al. 2012). In Magallanes, these interactions have been described in marine invertebrates inhabiting intertidal zones and rocky bottoms (e.g. Benedetti-Cecchi and Cinelli (1997), Lorenti and Mariani (1997), Prado and Castilla (2006), Andrade et al. (2016), Velasco‐Charpentier et al. (2021)). This region harbours approximately 50% of Chile’s mollusc biodiversity (Valdovinos 1999), a pattern attributed to the influence of ocean basins, which may shape mollusc distribution and diversity in the region (Valdovinos et al. 2003). Additionally, some species hold high commercial value for both local and international fisheries (Sánchez et al. 2011).

In the Strait of Magellan, records of benthic fauna remain sparse (Aldea et al. 2020), which is significant considering that Mollusca, one of the largest marine phyla, is a major component of this ecosystem (GBIF.org 2024). The Strait features an extensive and diverse coastline, with many areas dominated by rocky substrates that provide complex habitats for benthic communities (Ríos and Mutschke 1999). However, the lack of descriptive studies in certain areas, such as Cape Froward, has led to some localities being classified as low-diversity areas or lacking data despite their potential to support rich benthic assemblages (Aldea et al. 2020).

Cape Froward, where ice masses retreated from the fjords towards the Cordillera Darwin and southern ice fields, formed a proglacial lake that drained into the Pacific Ocean through the Famine Passage approximately 12,000 years BP (Mcculloch et al. 2005). Bougainville Bay, located south of Mansa Bay — one of the most diverse localities known in the Strait of Magellan (Aldea et al. 2020) — is an important intermediate point both in terms of glacial history and the socio-ecological context surrounding it.

This study aims to characterise the taxonomic composition of mollusc assemblages in the area and provide insights into their functional roles. Knowledge about their traits remains limited, yet as an important group of marine invertebrates, molluscs contribute to nutrient cycling, primary production and energy transfer within trophic networks.

## Materials and Methods

**Figure 1.**
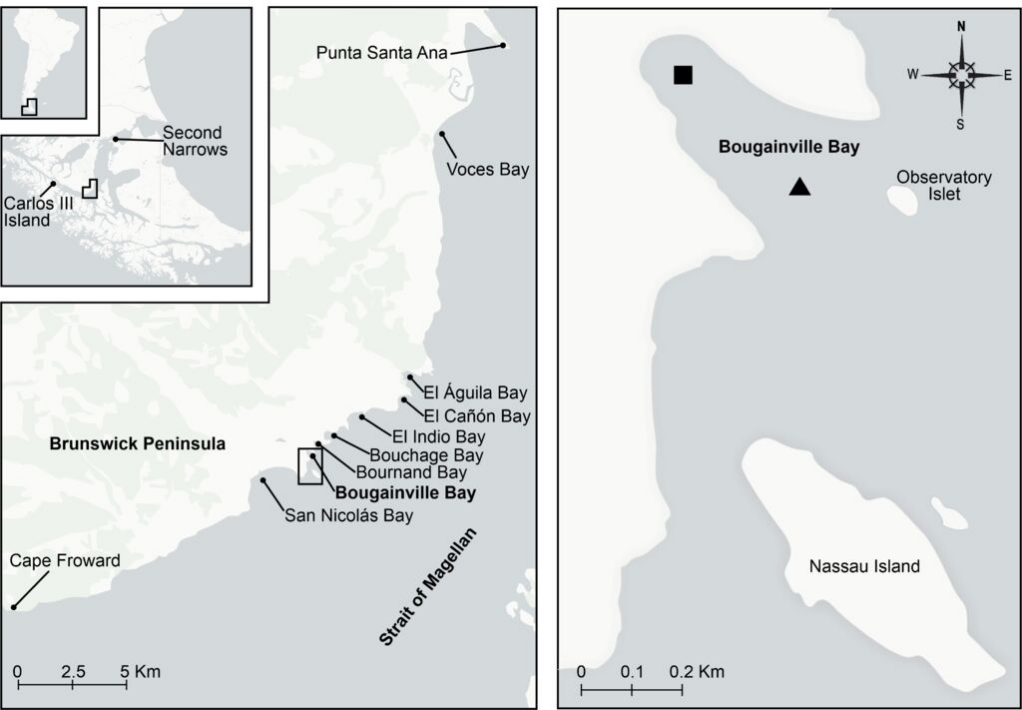
Study area of Bougainville Bay, Brunswick Peninsula, the southern tip of South America. The square indicates sampling in 2018 and the triangle indicates sampling in 2023.

Bougainville Bay is a small inlet (0.14 km²) located about 18 km northeast of Cape Froward (53°49'18"S, 71°04'24"W), precisely on the south-eastern margin of the Brunswick Peninsula (Fig. 1). From Punta Santa Ana towards Cape Froward, the coastline extends about 55 km along the Strait of Magellan. This coast, highly exposed to ship traffic, has a system of eight small bays (< ca. 4 km²; Fig. 1), some of which are used as ports by fishing vessels for temporary rest during navigation.

Sublittoral sampling was performed using two different methods: autonomous diving (20 m depth) in 2018 and a modified Agassiz trawl (44 m depth) in 2023. Both sampling efforts were conducted in February. The collected samples were preserved in 75% ethanol and taxonomically identified to the most specific level possible, based on morphological characteristics. This information was verified using published literature and databases, including GBIF: The Global Biodiversity Information Facility (2024) platform. All data obtained were standardised under the Darwin Core format (Wieczorek et al. 2012) and taxonomically adjusted according to the World Register of Marine Species (WoRMS Editorial Board 2025).

To compare the diversity of Bougainville Bay with other areas, we used Shannon’s species richness test (Spellerberg and Fedor 2003) and grouped the species into feeding guilds, based on their feeding strategies and diets. Guilds were defined by similarities in food particle size and composition, ingestion mechanisms and mobility patterns associated with feeding (Fauchald and Jumars 1979). For some species or genera, guild assignments were based on available literature for the Magellan Region. In cases where specific information was unavailable, classifications were inferred from closely-related taxa. Species were also categorised by trophic level, representing their position within the food chain. While this analysis is preliminary and specific to the study area, it provides a foundation for more detailed evaluations in future research.

## Results and Discussion

The biodiversity described in this study for Bougainville Bay consists of 49 operational taxonomic units (OTUs) with an H’ index of 3.413. All these OTUs are new records for this location (32 gastropods, nine bivalves and eight polyplacophorans), as no molluscan species have been previously recorded in Bougainville Bay. In addition, these occurrences represent new records for the neighbouring bays along an extensive coastline (~ 27 km) from Cape Froward to El Águila Bay (see Fig. 1), which previously shared only two documented species, *Zygochlamys patagonica* and *Philobrya sublaevis*. This information was cross-referenced with the GBIF database.

**Figure 2.**
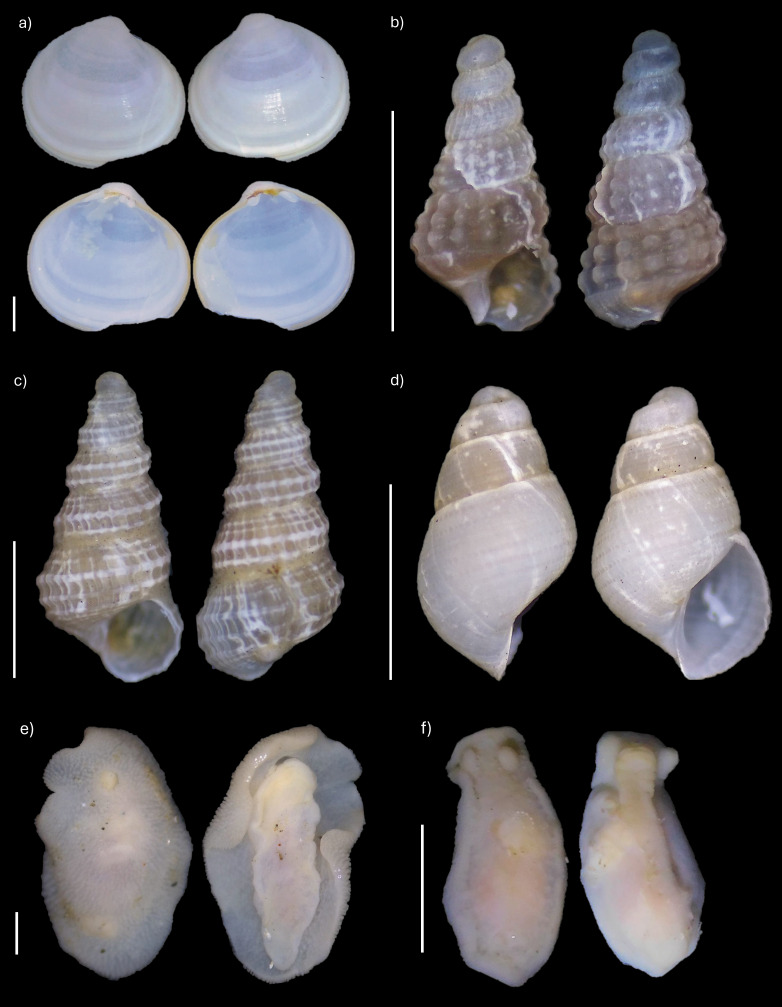
New species records in the central micro-basin of the Strait of Magellan: (a) Kellia cf. suborbicularis; (b) *Savatieria areolata*; (c) *Mathilda malvinarum*; (d) *Menestho beaglensis*; (e) *Dialula punctuolata*; (f) *Tyrinna delicata*. Scale bars: 1 mm (b, c, d, e), 2 mm (a, f).

**Figure 3.**
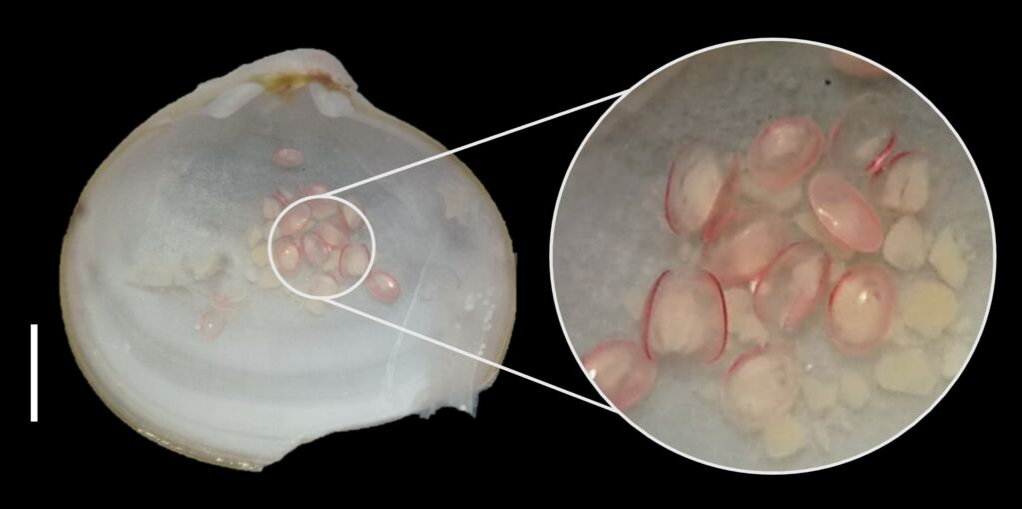
Kellia cf. suborbicularis and D-shell shaped larvae. Scale bar: 2 mm.

Regarding the central micro-basin of the Strait of Magellan, which extends from Second Narrows to Carlos III Island, this study presents six new records for the area (Aldea et al. 2020): one bivalve and five gastropods, including two nudibranchs (Fig. 2). However, after the revision by Aldea et al. (2020), *Mathilda malvinarum*, *Dialula punctuolata* and *Tyrinna delicata* were recorded in GBIF from specific collections. Amongst the samples observed, *Kellia suborbicularis* has not been recorded south of 43°S for approximately 100 years, according to the GBIF platform. In this study, juveniles were found within its interior (Fig. 3), as this organism hatches larvae in the gill lamellar cavity (Lebour 1938).

**Figure 4.**
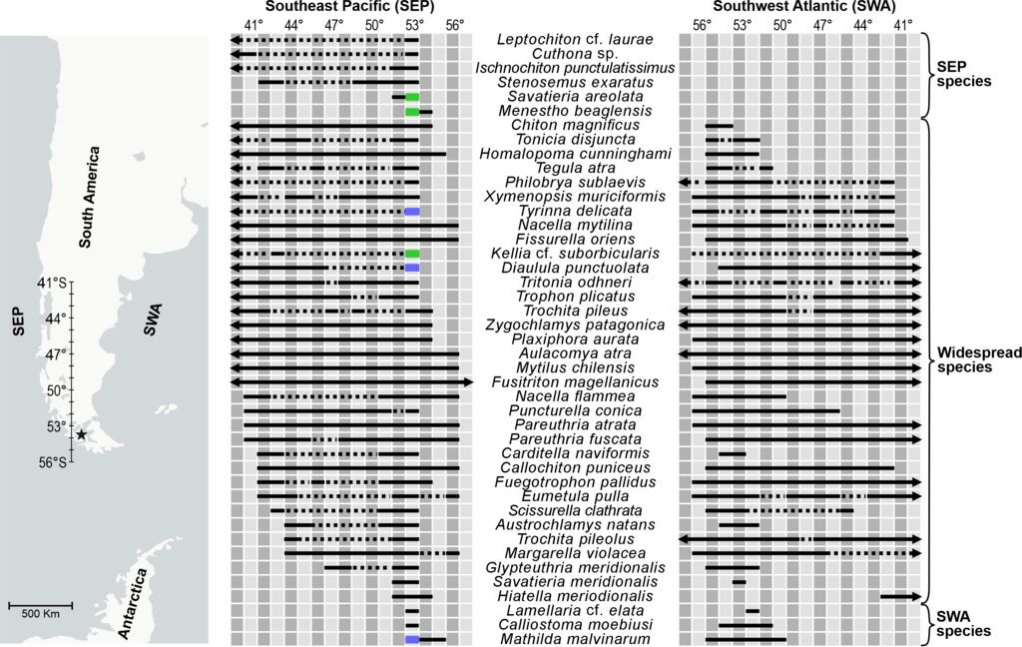
Distribution of the species recorded in Bougainville Bay (indicated with a star) from 41°S to 56°S on both the west and east sides of southern South America. Records that do not represent species (genus or higher levels) were not included, except for *Cuthona* sp. (new record for the central area of the Strait of Magellan). Arrows indicate the extended distribution towards lower or higher latitudes and dotted lines indicate absence of records in intermediate latitudes of the distribution. Green colours represent new records for this area and blue colours indicate records present in GBIF after Aldea et al. (2020), but not yet published in literature.

Analysing the species distribution, we observe that 79% of the species present in Bougainville Bay are found in both the Pacific and Atlantic Oceans. Amongst those not shared between these oceans, 14% occur in the Southeast Pacific and 7% in the Southwest Atlantic (Fig. 4).

**Figure 5.**
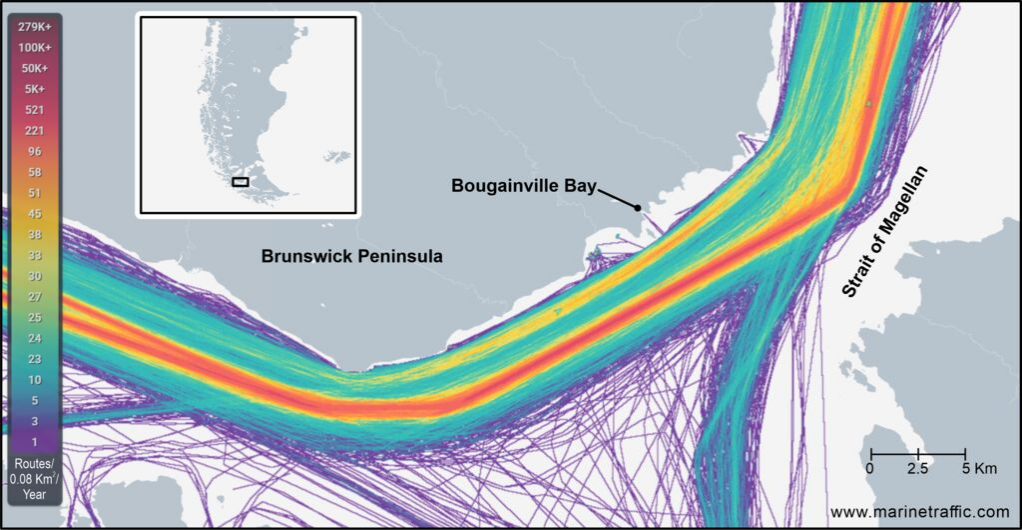
Marine traffic in the Strait of Magellan during 2023.

The glacial history of this Bay is of significant interest, as the Strait of Magellan was occupied by ice during multiple glacial cycles (Meglioli 1992, Rabassa et al. 2000). During the Last Glacial Maximum, the water level dropped approximately 120 m (Fairbanks 1989), exposing submerged geographic barriers, including the Second Narrows. Glaciers advanced northwards from the Cordillera Darwin (Darwin Mountain Range), forming an ice barrier that isolated the central region of the Strait of Magellan. Subsequently, glaciers retreated, creating extensive proglacial lakes between the ice front and the Second Narrows (Mcculloch and Bentley 1998), which directly influenced the surrounding environments. The drainage of these lakes occurred from the Atlantic to the Pacific (Mcculloch et al. 2005), which may be one of the factors influencing the composition of the benthic biota of marine molluscs in Bougainville Bay and its surrounding areas. These areas of high maritime traffic require further investigation, as they may represent cryptic sources of biodiversity (Fig. 5). This is particularly relevant given their proposed inclusion within the new Cape Froward National Park.

**Figure 6.**
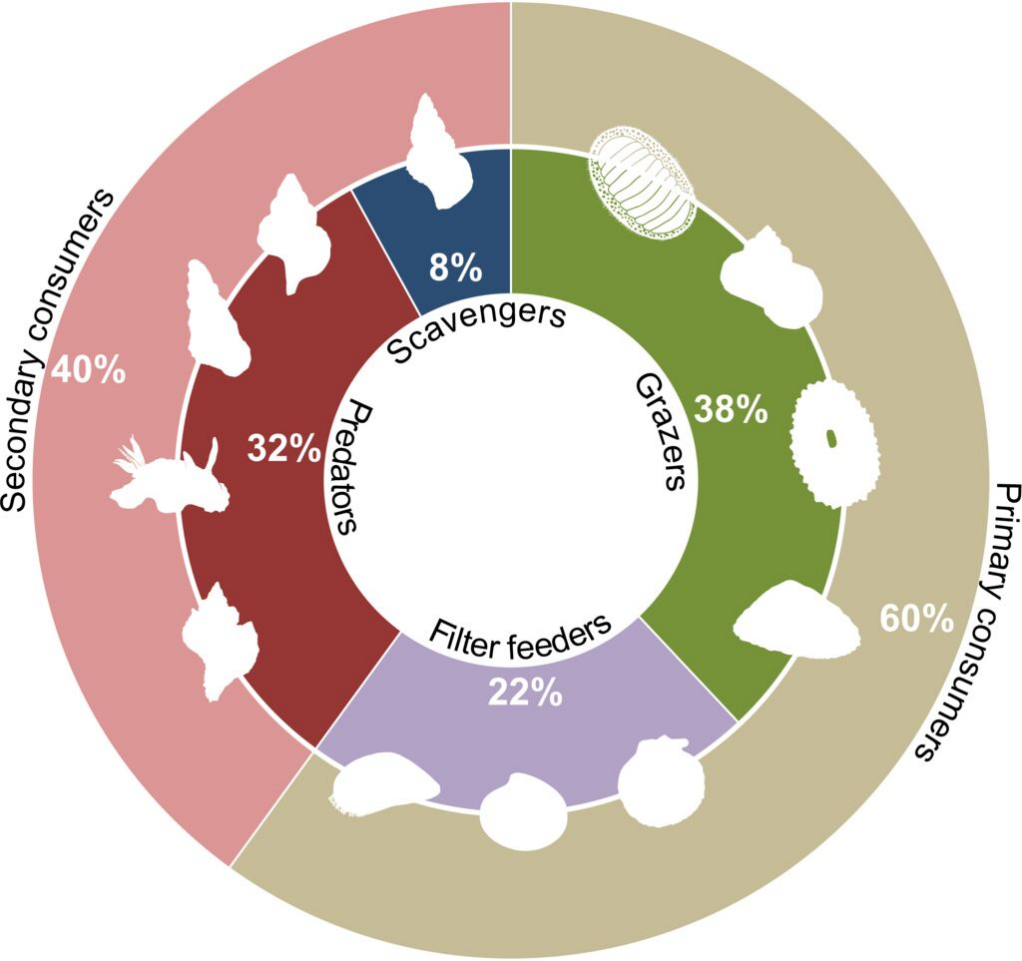
Contribution of mollusc feeding guilds (filter feeders, grazers, predators and scavengers) and trophic roles in Bougainville Bay.

The analysis of mollusc species collected in Bougainville Bay shows a broad representation of primary consumers (Fig. 6), including filter-feeding and grazing species, such as *Aulacomya atra*, *Mytilus chilensis* and *Nacella* spp. These species play a crucial role in filtering suspended particles and removing benthic algae, suggesting a significant contribution of molluscs to ecological stability by controlling primary biomass. Additionally, these species are the most dominant in terms of abundance in these rocky coastal areas, further highlighting their ecological importance (Ríos and Mutschke 1999).

In contrast, predators and scavengers, such as *Fuegotrophon pallidus*, *Tritonia* sp. and *Pareuthria* spp., were less abundant, representing secondary consumers (see Fig. 6). This difference may suggest a trophic structure dominated by primary consumers, potentially supporting a system where energy transfer is primarily driven by herbivory and filter-feeding processes. Notably, free-living gastropod grazers tend to be more prevalent at higher latitudes, as reported by Floyd et al. (2020), aligning with the high representation of herbivorous molluscs in this study. Additionally, Andrade et al. (2016) found that at least 50% of molluscs in similar environments are omnivores and grazers, reinforcing the idea that herbivory plays a key role in shaping benthic community dynamics in high-latitude ecosystems.

In this study, molluscs contribute to both primary and secondary trophic levels and, therefore, play an essential role in the local ecosystems of the Magellan region (Andrade et al. 2016). Understanding how ecosystems and species respond to climatic changes provides a broad perspective on both the ecology of organisms and the conservation biology of ecosystem assemblages (McCarty 2002, Marshall et al. 2008).

It is important to note that the new records presented in this work are essential for complementing knowledge of the biodiversity of molluscs inhabiting an area frequently used for maritime activities. These records also highlight how such areas can serve as indicators of anthropogenic impacts on coastal bays (Oehlmann and Schulte-Oehlmann 2003). Therefore, incorporating oceanographic, geographic and biological variables is necessary to better understand speciation processes in particular environments (Dawson et al. 2011).

## Data and material availability

All data from this study are available in the GBIF dataset *Biodiversity of Benthic Mollusca of Bougainville Bay (Strait of Magellan, Chile)*. The dataset includes 121 occurrences, representing three classes (Polyplacophora, Gastropoda and Bivalvia), 15 orders, 31 families and 49 OTUs (Aldea et al. 2025). All material was catalogued with the voucher “benthic-mollusks-bougainville-bay” and was deposited in the Austral Collection of Marine Invertebrates of the Universidad de Magallanes, Punta Arenas, Chile.
